# Prevalence and risk factors of mood and anxiety disorders in children and young people: Findings from the Northern Ireland Youth Wellbeing Survey

**DOI:** 10.1177/13591045221089841

**Published:** 2022-05-18

**Authors:** Lisa Bunting, Emma Nolan, Claire McCartan, Gavin Davidson, Anne Grant, Ciaran Mulholland, Dirk Schubotz, Orla McBride, Jamie Murphy, Mark Shevlin

**Affiliations:** 1 Queen’s University Belfast, Belfast, Northern Ireland; 2 Ulster University, Coleraine, Northern Ireland

**Keywords:** Northern Ireland Youth Wellbeing Survey (NIYWS), child and adolescent, mood and anxiety disorders

## Abstract

**Objective:**

This paper presents the key findings from the Northern Ireland Youth Wellbeing Prevalence Survey (NIYWS), specifically the prevalence of common mental health disorders and their association with personal, familial and socio-economic risk factors.

**Methods:**

The Northern Ireland Youth Wellbeing Survey (NIYWS) is a large nationally representative household survey of young people aged 2–19 years (N = 3074) and their parents (N = 2816). Data collection was by means of a stratified random probability household survey. Children and young people were eligible to take part if they were aged 2 to 19 and lived in Northern Ireland. Mood and anxiety disorders were measured using the Revised Children’s Anxiety and Depression Scale (RCADS: Chorpita et al., 2000).

**Results:**

Based on the cut-off scores for the RCADS 11.5% of the sample met the criteria for any mental health disorder. The most prevalent disorder was panic disorder (6.76%) and the least common was generalised anxiety disorder (2.69%). Poor child health, special educational needs, parental separation, living in a household in receipt of benefits, living in an area of deprivation and living in an urban area were all significant predictors of any mood or anxiety disorder.

**Conclusions:**

The results indicate somewhat elevated prevalence rates of mood and anxiety disorders in children and young people in Northern Ireland compared to England and other international countries. These findings can be used to help inform mental health policy and practice.

## Introduction

Recurring surveys in both the UK and USA, (McManus et al., 2020; Kessler et al., 2005), as well as surveys conducted under the auspices of the World Mental Health Survey Initiative (Kessler et al., 2006), provide reliable and valid estimates of the prevalence of mental health disorders in adult populations across numerous countries. Although surveys of child and adolescent mental health are less common, various countries, including the US, England and Ireland, also benefit from nationally representative surveys investigating the prevalence of mental health problems amongst their child and/or adolescent populations (see, for example, Kessler et al., 2009; Sadler et al., 2018; and Dooley et al., 2019). Worldwide, pooled prevalence estimates of common mental health disorders amongst children and adolescents are 2.6% (CI; 95% 1.7–3.9) for any depressive disorder and 6.5% (CI 95% 4.7–9.1) for any anxiety disorder (Polanczyk et al., 2015), The Mental Health of Children and Young People Survey (MHCYPS) has been providing prevalence rates of mental health problems for young people in the UK since 1999, with the 1999 and 2004 surveys including children from England, Scotland and Wales and the 2017 survey focusing on England only (Ford et al., 2020). The 2017 survey found that one in eight (12.8%) 5–19 year olds had at least one clinically diagnosable mental health disorder, with one in 12 (8.1%) having an emotional disorder such as anxiety or depression and one in 20 (4.6%) having a behavioural or ‘conduct’ disorder (Sadler et al., 2018). Comparison of the 2017 results with those from previous years suggested a slight increase in overall mental health disorder rates over time. However, a 2020 follow up focusing on more general mental health functioning pointed to more substantial increases with the proportion of 5 to 16 year olds with a probable mental health disorder rising from 11.4% in 2017 to 16.7% in 2020 (Vizard et al., 2020). While these increases are likely attributable to the COVID-19 pandemic (research has shown that younger peoples’ mental health has been disproportionately affected by the pandemic; Shevlin et al., 2020), research with Irish adolescents conducted pre-pandemic has also reported increases of 7% and 11% amongst those classified within the severe range for depression and anxiety, respectively, since 2012 (Dooley et al., 2019). Similar longitudinal increases in the rates of youth mental health problems have also been observed in the international literature (Collishaw & Sellers, 2020).

In the face of high, and increasing, levels of the mental health problems reported by young people internationally, as well as in the UK and Ireland, there are no epidemiological data on mental health disorders in children and young people in Northern Ireland (NI). This is despite the fact that research with the NI adult population has identified a prevalence estimate of common mental health disorders of 39.1%, one of the highest in Western Europe (Bunting et al., 2012). Although linked to a variety of factors, the legacy of political violence (Cummings, Goeke-Morey, Shermerhorn, Merrilees & Cairns, 2009) is one of the most commonly cited explanations for these elevated rates. Greater exposure to the ‘Troubles’ (a 30 year period of political violence in Northern Ireland, ending in 1998 that resulted in over 3500 deaths) has been shown to be associated with increased levels of mental health problems in later life (O'Reilly & Stevenson, 2003), and NI has been shown to have the highest rate of PTSD of any of the 30 countries which participated in the World Mental Health Survey Initiative (Bunting et al., 2013). While children and young people growing up in NI today will have had no direct experience of the ‘Troubles’, it is argued that having a parent with a mental health disorder, specifically those with PTSD resulting from exposure to the ‘Troubles’, potentially places younger generations at increased risk of developing a mental health disorder (Kelso, 2017).

In addition to trauma exposure, high rates of poverty (on a mixed measure of income and deprivation), educational inequality and unemployment are also commonly cited explanatory factors for the high levels of mental health problems in the NI adult population (Horgan & Monteith, 2009). While each constituent country of the UK has developed slightly different measures of area-level deprivation, comparisons based on the domains of employment and income, show higher levels of deprivation in NI than the rest of the UK (Abel et al., 2016). Equally, although household poverty, defined as those whose income is less than 60% of the median income, is slightly lower in NI than England or Wales, NI has a higher proportion of households without work, lower employment rates, more people with no qualifications and fewer people with higher level qualifications, than elsewhere in the UK (Barnard, 2018). While likely due to the combined effect of both the ‘Troubles’ and various economic downturns over the past two decades, it has been argued that this economic inequality has intensified the effect of the trauma-related mental health in the NI population (O'Connor & O'Neill, 2015).

Subsequently, in 2019 a nationally representative mental health survey of children and young people in Northern Ireland was commissioned by the Health and Social Care Board (NI). The primary aim was to provide robust and reliable evidence on the current prevalence of mental health problems and associated personal, familial and socio-economic risk factors to assist policy makers and service providers with future planning and development. This paper presents the key findings from the Northern Ireland Youth Wellbeing Prevalence Survey (NIYWS), specifically the prevalence of common mental health disorders and their association with personal (age, gender, physical health and special educational needs), familial (parental separation and parental mental health) and socio-economic (receipt of benefits, area-level deprivation and urbanicity) risk factors. These risk factors were selected to be consistent with other UK and Republic of Ireland mental health surveys of young people (Sadler et al., 2018; Donnelly et al., 2019).

## Method

### Sampling and data collection

The Northern Ireland Youth Wellbeing Survey (NIYWS) is a stratified random probability household survey undertaken by a consortium comprised of researchers from Queens University Belfast, Ulster University and members of the Northern Ireland Health and Social Care Board (Bunting et al., 2020, 2022). Children and young people were eligible to take part if they were aged 2 to 19 and lived in Northern Ireland. To produce reliable estimates of mental disorder prevalence, based on a population prevalence of 7.5% (the lifetime prevalence of psychotic-like experiences: McGrath et al., 2015) with a confidence level of 95% and precision of 1%, a target sample size of 2596 children and young people was identified.

Due to caution about the possible implications of the UK General Data Protection Regulation (2018) the research team were not given permission to access the NHS Patient Register, Child Benefit Register or other data registers that could have reliably indicated households with children eligible to participate in the study. As a result, it was necessary to randomly select addresses from households across Northern Ireland using the Pointer Database (a postcode register of all households in Northern Ireland) and, at the fieldwork stage, identify a household as either eligible or ineligible (eligibility based on a child aged 2 to 19 years residing in the property), following a visit from one of the interviewing team. Based on a conservative estimate that 1 in 5 households in NI had a resident child aged 2 to 19 years (Northern Ireland Statistics and Research Agency, 2012), and assuming a response rate of 50%, an initial sample of 30,000 was identified as necessary to achieve a final sample of 3000 completed interviews

In total, 762, 264 eligible addresses were identified and linked to Northern Ireland’s Multiple Deprivation Measures data (NIMDM; Northern Ireland Statistics and Research Agency, 2017) and stratified by deprivation decile and county to ensure even geographical distribution and representation of both affluent and less affluent neighbourhoods. A total of 30,000 addresses were then randomly selected and issued to the data collection team in six instalments from June 2019 to February 2020, and then clustered according to Electoral Ward to allow for a more efficient fieldwork process. The end of the fieldwork coincided with the onset of the COVID-19 global pandemic and ensuing national lockdown, at which point 21,730 main sample addresses had been issued

### Participants

In total 3074 surveys were completed giving a response rate of 67%. The final sample closely matched the NI population in terms of gender, age, geographical location and deprivation (see Bunting et al. 2022, for a detailed description of the survey methodology, sample characteristics and measures used).

### Measures

Mood and Anxiety Disorders: Mood and anxiety disorders were measured using the Revised Children’s Anxiety and Depression Scale (RCADS; Chorpita et al., 2000). The RCADS is a 47-item questionnaire that produces indications of clinically relevant levels of severity of six disorders derived from the diagnostic criteria of the DSM-IV (American Psychiatric Association, 2000): major depressive disorder (MDD), separation anxiety (SAD) disorder, social phobia (SP), generalised anxiety disorder (GAD), panic disorder (PD) and obsessive compulsive disorder (OCD). One of the more widely used brief screening instruments for symptoms of anxiety and depression, RCADS has shown robust internal consistency reliability in different assessment settings, countries and languages (Piqueras et al., 2017), good test–retest reliability (Chorpita et al., 2000) and good convergent validity (Esbjørn et al., 2012; Bouvard et al., 2015). The six subscales yield sensitivity scores ranging from 0.59–0.78 and specificity scores ranging from 0.64–0.92, with the MDD scale and PD scale showed the most favourable predictions (Chorpita et al., 2005). Importantly, it has shown good reliability and validity within a population of Irish youth aged 12–18 years (Donnelly et al., 2019).

The scale is available in formats that can be self-completed or completed by a parent/carer; the parent version has been validated for use with children aged 3–17 years (Ebesutani et al., 2015). In this study, parents of 0–10 years olds completed the parent version and 11–19 year olds completed the self-report version. Each item is scored on a 4-point Likert response scale (0 =never to 3 = almost always) and raw subscale scores are converted into t-scores which are normed based on school year and gender. This process is facilitated using syntax available from the developer (https://www.childfirst.ucla.edu/resources). The rates for ‘clinical’ levels are reported. The reliability estimates (Cronbach’s α) for all the subscale scores were acceptable: major depressive disorder (α = .89), separation anxiety disorder (α = .77), social phobia (α = .92), generalised anxiety disorder (α = .89), panic disorder (α = .90) and obsessive compulsive disorder (α = .83).

Age and Gender: For 0–10 year olds, age (years) and gender (Male, Female, Other, Prefer not to say) were reported by the parents/carer and self-reported for 11–19 year olds. Age was recoded into a four-category variable (2–4 years, 5–10 years, 11–15 years and 16–19 years; see Table 1) and a 2-category variable (2–10 years and 11–19 years; see Table 2).

**Table 1.** Sample Demographics for Participants from the Northern Ireland Youth Wellbeing Survey.

Characteristics	All participants (N = 3074)	
**Age, Mean (SD)**	15.22 (2.58)	
	**N**	**%**
**Age categories (Years)**		
2–4	641	20.9
5–10	1134	36.9
11–15	670	21.8
16–19	629	20.5
**Gender**		
Male	1590	51.7
Female	1477	48.1
Other/prefer not to say		
**Ethnicity**		
White	2680	95.2
Irish traveller	8	0.3
Chinese	4	0.1
Indian	20	0.7
Pakistani	5	0.2
Bangladeshi	3	0.1
Black African	12	0.4
Black other	1	0.0
Mixed	18	0.6
Other	64	2.3
**Religious denomination**		
Roman Catholic	1213	39.5
Presbyterian	442	14.4
Church of Ireland	265	8.6
Methodist	57	1.9
Other	197	6.4
None	520	16.9
Prefer not to say	122	4.0
**Urban/Rural**		
Urban	1831	59.6
Rural	922	30.0
Mixed	321	10.4
**County**		
Antrim	1085	35.3
Armagh	271	8.8
Down	925	30.1
Fermanagh	122	4.0
Londonderry	431	14.0
Tyrone	240	7.8

**Table 2.** Prevalence Estimates (95% confidence intervals) of RCADS Disorders Stratified by Child, Family and Social Factors.

	Depression	GAD	OCD	Panic	Separation	Social	Any dx
**Total**	5.01 (4.22–5.80)	2.69 (2.10–3.27)	3.10 (2.47–3.73)	6.76 (5.85–7.67)	5.17 (4.37–5.98)	3.75 (3.03–4.43)	11.50 (10.34–12.65)
**Gender**							
Male	5.46 (4.31–6.61)	3.12 (2.24–4.01)	3.33 (2.42–4.24)	7.55 (6.21–8.89)	4.93 (3.83–6.02)	3.46 (2.54–4.39)	11.84 (10.21–13.48)
Female	4.53 (3.46–5.61)	2.23 (1.46–2.99)	2.85 (1.99–3.72)	5.93 (4.79–7.15)	5.43 (4.26–6.61)	4.04 (3.02–5.06)	11.13 (9.51–12.76)
**Age**							
2–4	0.48 (0.06–1.01)	0.16 (0.15–0.47)	0.48 (0.06–1.01)	1.11 (0.29–1.93)	0.63 (0.01–1.26)	0.16 (0.15–0.47)	2.06 (0.95–3.17)
5–10	5.85 (4.48–7.22)	3.72 (2.62–4.83)	3.73 (2.62–4.83)	6.75 (5.28–8.22)	6.20 (4.79–7.61)	4.61 (3.38–5.84)	12.04 (10.14–13.94)
11–15	5.22 (3.43–7.01)	3.02 (1.64–4.39)	2.35 (1.13–3.57)	7.74 (5.59–9.90)	5.19 (3.41 6.98)	3.52 (2.04–5.00)	12.90 (10.20–15.59)
16–19	8.12 (5.89–0.35)	3.10 (1.69–4.51)	5.53 (3.66–7.39)	1.94 (9.29–4.59)	8.12 (5.89–0.35)	6.22 (4.24–8.19)	19.28 (16.06–22.49)
**Special educational needs**							
No	3.17 (2.50–3.84)	1.91 (1.38–2.43)	1.98 (1.45–2.52)	5.05 (4.21–5.89)	3.59 (2.88–4.31)	2.75 (2.12–3.38)	8.71 (7.63–9.79)
Yes	20.06 (15.66–24.45)	9.09 (5.93–12.24)	12.26 (8.65–15.87)	20.82 (16.35–25.29)	18.06 (13.86–22.27)<	11.91 (8.35–15.46)	34.27 (29.05–39.49)
**Child health**							
Very Good/Good	3.06 (2.41–3.71)	1.94 (1.41–2.46)	2.24 (1.68–2.80)	4.68 (3.87–5.48)	3.65 (2.94–4.37)	2.76 (2.14–3.38)	8.77 (7.69–9.84)
Fair/Bad/Very Bad	27.31 (21.51–33.10)	11.30 (7.21–15.39)	13.59 (9.14–18.04)	30.70 (24.71–36.68)	23.24 (17.76–28.72)	14.97 (10.33–19.62)	42.61 (36.17–49.05)
**Parental separation**							
Living with both biological parents	3.943 (3.09–4.79)	2.267 (1.61–2.91)	2.467 (1.71–3.14)	5.531 (4.53–6.52)	4.438 (3.54–5.33)	3.107 (2.31–3.86)	9.80 (8.50–11.09)
Not living with both biological parents	7.46 (5.73–9.19)	3.71 (2.46–4.95)	4.51 (3.14–5.88)	9.38 (7.46–11.31)	6.99 (5.31–8.66)	5.30 (3.82–6.78)	15.30 (12.93–17.67)
**Parental mental health problems**							
No	3.29 (2.52–4.06)	1.74 (1.17–2.30)	2.03 (1.42–2.64)	4.90 (3.97–5.83)	3.43 (2.65–4.22)	2.37 (1.71–3.03)	7.89 (6.72–9.05)
Yes	7.64 (10.14–12.63)	4.23 (6.22–8.22)	4.38 (6.41–8.44)	9.58 (12.29–15.01)	8.60 (11.21–13.81)	5.77 (8.02–10.26)	21.85 (18.42–25.27)
**Family benefits**							
No benefits	3.12 (2.33–3.91)	1.61 (1.03–2.18)	2.15 (1.49–2.81)	4.90 (3.91–5.88)	3.81 (2.94–4.69)	2.63 (1.90–3.36)	9.99 (8.64–11.33)
Benefits	8.28 (6.63–9.93)	4.55 (3.31–5.80)	4.74 (3.47–6.02)	9.99 (8.19–11.78)	7.52 (5.95–9.10)	5.68 (4.29–7.07)	16.69 (14.55–18.84)
**Multiple deprivation**							
Quintiles 2–5	4.56 (3.71–5.39)	2.49 (1.86–3.11)	3.08 (2.38–3.78)	6.05 (5.08–7.00)	4.68 (3.83–5.53)	3.55 (2.80–4.29)	10.66 (9.41–11.90)
Quintile 1 (Most deprived 20%)	6.92 (4.83–9.02)	3.53 (2.01–5.05)	3.18 (1.73–4.63)	9.76 (7.31–12.22)	7.25 (5.11–9.39)	4.60 (2.87–6.32)	15.02 (12.07–17.97)
**Living location**							
Rural/Mixed	2.95 (1.98–3.91)	1.85 (1.08–2.62)	2.36 (1.49–3.23)	4.81 (3.59–6.03)	3.37 (2.34–4.39)	2.45 (1.57–3.33)	8.59 (7.00–10.19)
Urban	6.41 (5.26–7.56)	3.25 (2.42–4.08)	3.60 (2.73–4.47)	8.08 (6.80–9.36)	6.40 (5.25–7.55)	4.62 (3.64–5.61)	13.46 (11.86–15.06)

Special Educational Needs: Parents/carers of children aged 0–10 years were asked ‘*Does your child have a diagnosed or suspected special educational need*’*?* and participants aged 11–19 were asked ‘*While at school, did you ever have a diagnosed or suspected special educational need*’*?* The response options were ‘Yes’, No’ and ‘Prefer not to say’ and these were recoded to 1 (Yes) and 0 (No or Prefer not to say).

Child Health: Parents/carers of 0–10 year olds were asked ‘*How is your child’s health in general? Would you say it was…*’ and participants aged 11–19 were asked ‘*How is your health in general? Would you say it is…*’ and the possible responses were (1) Very Good, (2) Good, (3) Fair, (4) Bad and (5) Very Bad. These scores were dichotomised to represent poor physical health (1= Bad or Very Bad, 0 = Very Good, Good or Fair)

Parental Separation: Participants were considered to be living with both biological parents if adult respondents answered ‘Yes’ to ‘*Are you living in this household with a partner*’ and selected the option ‘Parent’ to both questions ‘*What is your relationship to the? nominated child*’ and ‘*What is your partner’s relationship to the nominated child*’*?* Where parents did not complete the parent survey, participants aged 16–19 years were asked ‘*Are your parents*. . .’ and were provided with options ‘Married’, ‘Living with a partner as if married’, ‘Single (never married)’, ‘Separated’, ‘Divorced’ or ‘Widowed’. They were considered to be living with both biological parents if they selected ‘Married/Living together as if married’.

Parental Mental Health: The General Health Questionnaire (GHQ-12: Goldberg & Williams, 1988) was used to assess current mental health status among parents. The GHQ-12 is a widely used screening measure for identifying psychiatric morbidity in the general population (McManus et al., 2016). It is a 12-item self-completion questionnaire comprised of statements reflecting symptoms common in mood and anxiety disorders (e.g., ‘*Have you recently been feeling unhappy and depressed*’*?* ‘*Have you recently lost much sleep over worry*’*?*). The response options were ‘Not at all, No more than usual, Rather more than usual, Much more than usual’, and were scored using 0, 0, 1 and 1, respectively, as recommended by Goldberg and Williams (1988). This process produced scores ranging from 0 to 12 and a cut-off score of 4 or more was used to identify individuals with potential mental health problems.

Family Benefits: Parents/carers and young people over the age of 16 were asked ‘*Is your household receiving any of these state benefits*’? and provided with a list of state benefits and asked to indicate which they were in receipt of (Universal Credit/Housing Benefit/Working Tax Credit/Child Tax Credit/Income support/Jobseeker’s Allowance/Employment and Support Allowance/Carer’s Allowance/Disability Living Allowance/Personal Independence Payment). If none were selected the family was coded as ‘No Benefits’.

Multiple Deprivation: Deprivation was measured using Northern Ireland Multiple Deprivation Measure 2017 (NIMDM; Northern Ireland Statistics and Research Agency, 2017) scores. The NIMDM measures deprivation on seven domains: health; income; employment; education skills and training; proximity to services; living environment; and crime and disorder. Weighted scores are derived by calculating the number of people experiencing each type of deprivation in a Super Output Area (SOA). The SOA for each residence was recorded and then linked to Northern Ireland’s 2017 NIMDM data and stratified by deprivation decile. This was further recoded to identify the top 20% areas of deprivation.

Living Location: Northern Ireland Statistics and Research Agency (NISRA) calculates ‘Settlement band’ (https://www.nisra.gov.uk/publications/settlement-2015-documentation) as an index that places all settlements in Northern Ireland on an urban-rural spectrum according to population size, population density and service provision. There are currently eight settlement bands (A-H) on this spectrum: (1) Belfast metropolitan urban area, (2) Derry urban area, (3) large town, (4) medium town, (5) small town, (6) intermediate settlement, (7) village and (8) small village, hamlet or open countryside. This variable was collapsed to a three category variable: Urban (Bands A-B), Mixed (Bands C-G) and Rural (Band H).

### Data Analysis

Prevalence of RCADS disorders and associated 95% confidence intervals were calculated, and a variable was created to indicate if a participant meet the criteria for any disorder or not. This variable was then used in cross tabulations to calculate the counts and percentages when stratified by the risk factors. Bivariate binary logistic regressions were used to estimate the odds ratios (ORs) associated with each risk factor, and then a multivariate model was used with all the predictors entered in the same model to estimate adjusted ORs. This approach provided unadjusted and adjusted estimates of association, the latter indicating the associations when all other variables were controlled for. The ORs indicate the predicted increase, or decrease, in the likelihood of ‘any disorder’ for each of the predictors. The statistical significance of the ORs was assessed using 95% confidence intervals: if the value ‘1’ lies below the lower confidence interval or above the upper confidence interval, this represents statistical significance at the .05 level. The average amount of missing data missing data across all variables was low (2.65%) and this was handled using full information maximum likelihood estimates of the regression models as recommended by Schafer and Graham (2002).

## Results

The Northern Ireland Youth Wellbeing Survey (NIYWS) recruited a large nationally representative household survey of young people aged 2–19 years (N=3074) and their parents (N=2816). Descriptive statistics for demographic variables are presented in Table 1. The majority of the sample were white (95.2%), with gender approximately equal (51.7% male) and 42.3% aged 11–19 years. The most commonly reported religious denomination was Catholic, and over half resided in an urban area (59.6%).

Table 2 shows the prevalence rates for each of the RCADS disorders, stratified by demographic and social factors. Based on the cut-off scores for the RCADS, around 11.5% met the criteria for any mental health disorder. The most prevalent disorder was Panic Disorder (6.76%, 95% CI 5.85–7.67) and the least common was generalised anxiety disorder (2.69, 95% CI 2.10–3.27). Poor child health and child special educational needs were associated with higher prevalence of RCADS disorders, while parental separation, living in a household in receipt of benefit, living in an area of deprivation and living in a urban area also produced higher estimates. Males had consistently higher prevalence estimates compared to females across all RCADS disorders, with the exception of Social Phobia, Similarly, young people in the oldest age category (16–19 years), had consistently higher prevalence estimates across all RCADS disorders, with the exception of panic disorder, with 19.3% having an RCADS disorder. The most prevalent disorder for the 2–4, 5–10 and 11–15 year age groups was panic disorder, for the 16–19 age group it was depression and separation anxiety, respectively.

Further examination of age and gender groupings produced higher prevalence estimates for depression and anxiety among 5–10 year old boys compared 5–10 year old girls (depression = 8.4% vs 2.9%; anxiety = 7.6% vs 3.2%), as well as 16–19 year old girls compared to boys (depression = 10.7% vs 5.4%; anxiety = 11.7% vs 8.1%).

Table 3 shows the percentages and the unadjusted and adjusted odds ratios (95% confidence intervals, CI) of any RCADS disorder across the social, familial and demographic predictors. At the bivariate level, older age, special educational needs, parental separation, parental mental health problems, being receipt of family benefits, living in an area of high deprivation and living in an urban area were all associated with higher prevalence estimates of any RCADS disorder. Child gender was not associated with higher prevalence estimates of any RCADS disorder.

**Table 3.** Bivariate and Multivariate Binary Logistic Regression Results Predicting Any RCADS Disorder.

		Any RCADS	Unadjusted OR (95% confidence interval)	Adjusted OR (95% confidence interval)
	N	N (%)		
Age				
2–10 years	1775	149 (8.5%)	—	—
11–19 years	1299	189 (16.0%)	2.06* (1.64–2.60)	2.05* (1.59–2.63)
Gender				
Male	1590	178 (11.8%)	—	—
Female	1477	160 (11.1%)	0.93 (0.74–1.17)	0.99 (0.77–1.27)
Special educational needs				
No	2690	228 (8.7%)	—	—
Yes	384	110 (34.3%)	5.46* (4.18–7.14)	4.54* (3.36–6.12)
Child health				
Very Good/Good	2747	235 (8.8%)	—	—
Fair/Bad/Very Bad	249	98 (42.6%)	7.72* (5.76–10.36)	4.87* (3.48–6.81)
Parental separation				
Living with both biological parents	2095	199 (9.8%)	—	—
Not living with both biological parents	958	136 (15.3%)	1.66* (1.32–2.11)	1.02 (0.76–1.36)
Parental mental health problems				
No	2146	163 (7.9%)	—	—
Yes	607	123 (21.8%)	3.24* (2.52–4.19)	2.37* (1.77–3.18)
Family benefits				
No benefits	1912	162 (8.7%)	—	
Benefits	1162	176 (16.4%)	2.05* (1.64–2.58)	1.52* (1.14–2.01)
Multiple deprivation				
Quintiles 2–5	2472	253 (10.7%)	—	—
Quintile 1 (Most deprived 20%)	602	85 (15.0%)	1.48* (1.14–1.93)	1.05 (0.77–1.43)
Living location				
Rural/mixed	1243	102 (8.6%)	—	—
Urban	1831	236 (13.5%)	1.65* (1.30–2.11)	1.35* (1.01–1.79)

Note: * *p* < .05.

The multivariate estimates reported in Table 3 show that older age, having special educational needs, parental mental health problems, being receipt of family benefits and living in an urban area, all retained significant associations with higher prevalence estimates of any RCADS disorder. Child health disorder (OR=4.87, CI - 3.48–6.81) and special educational needs (OR=4.54, CI - 3.36–6.12) had the strongest effects, resulting in an approximately a five-fold increase in the odds of experiencing any RCADS disorder. Child age retained a similar effect (OR=2.05, CI - 1.59–2.63), parental mental health problems remained significant with a muted effect (OR=2.37, CI - 1.77–3.18), as did being in receipt of benefits (OR= 1.52, CI - 1.14–2.01) and living in an urban area (OR=1.35, CI - 1.01–1.79). Finally, bivariate effects for not living with both biological parents and living in the 20% most deprived area not hold in the multivariate model and were no longer significant.

## Discussion

This study presents the initial findings from NIYWS, the first study to assess the prevalence of mood and anxiety disorders in a large representative youth sample from Northern Ireland. The primary aim of this study was to identify the rates of common mood and anxiety disorders and their association with personal, familial and social risk factors. The findings indicate around 11.5% of NI youth have a common mood or anxiety disorder, with 5% having a depressive disorder and 10.2% some form of anxiety disorder. Overall, panic disorder was the most common disorder (6.8%), followed by separation anxiety disorder (5.2%), depression (5.0%), social anxiety disorder, OCD (3.1%) and GAD (2.7%).

The NIYWS rates are somewhat higher than the worldwide pooled prevalence estimates of 2.6% (CI; 95% 1.7–3.9) for any depressive disorder and 6.5% (CI; 95% 4.7–9.1) for any anxiety disorder (Polanczyk et al., 2015), as well as the prevalence estimates of emotional disorder identified in the MHCYPS 2017 England (Vizard et al., 2018). However, in keeping with the international literature, anxiety disorder prevalence estimates were higher than depressive disorder estimates in both the NIYWS and MHCYP, with NIYWS estimates being higher than MHCYP rates across both domains (any depressive disorder, 5.0% vs 2.1%; any anxiety disorder, 10.7% vs 7.2%), as well as for each of individual disorders measured.

The literature commonly identifies higher rates of both depression and anxiety amongst females, with older teenagers typically having the highest rates (Hamblin, 2016). Similarly, while there was no overall significant gender difference in this study, further disaggregation by age and gender highlighted higher estimates of depression and anxiety among 16–19 year old girls compared to 16–19 year old boys (depression = 10.7% vs 5.4%; anxiety = 11.7% vs 8.1%). There were also substantially higher estimates of depression and anxiety among 5–10 year old boys compared 5–10 year old girls (depression = 8.4% vs 2.9%; anxiety = 7.6% vs 3.2%). Although the MHCYPS England (Sadler et al., 2018) has reported somewhat higher rates of emotional disorders among 5–10 year old boys compared to girls (4.6% vs 3.6%), the difference between the two groups was much more notable in the NIYWS. That this findings was also supported by data from other measures of emotional problems used within the NIYWS (Bunting et al.,2022), suggests it is not merely an artefact of methodological differences but reflective of different gender and age related patterns of prevalence among the NI youth population that warrant further consideration.

The data for this analysis were stratified based on personal, family and social factors, and significant associations between mental health problems and these risk factors were found. In relation to personal factors, meeting the criteria for any RCADS disorder was significantly associated with older age (11–19 years, OR = 2.05), special educational needs (OR = 4.54) and poor health (OR = 4.87). This reflects findings from the extant literature which consistently identifies higher rates of rates of mental health disorders amongst older children (Kessler et al., 2007), as well as associations between child mental health problems, physical health, chronic illness and disabilities (Pinquart & Shen, 2011a; 2011b; Augestad, 2017; Jiang et al., 2020). Likewise, the MHCYPS (2017) England found that older children, those with special educational needs and those with poorer health, had higher rates of emotional disorders than those without (Vizard et al., 2018).

While bivariate associations for certain familial and social factors, such as not living with biological parents and living in an area of deprivation had moderate effects in the bivariate analysis, these associations did not survive the multivariate model. However, in the adjusted model, having a parent with a mental health condition (OR = 2.37) and living in a household in receipt of benefits (OR = 1.52) both retained significant associations with any mood or anxiety disorder. Again, this mirrored the MHCYPS (2017) England which established higher emotional disorder rates among children whose parents had mental health problems (OR = 2.48) and those who were in receipt of both income (10.3% vs 5.9%) and disability based benefits (16.8% vs 6.0%), but found no significant associations with either parental marital status or area-level deprivation (Vizard et a., 2017). Living in an urban area was also significantly associated with common mood and anxiety disorders in the NIYWS sample (OR = 1.35), a finding supported by research with the NI adult population (OR = 1.65; Maguire & O’Reilly, 2015), as well as wider epidemiological research (Gruebner et al., 2017).

The NIYWS is the first ever survey measuring the mental health of children and adolescents in Northern Ireland. Its methodology and high response rate create a large sample (N=3074) that is representative of children across NI in terms of age and sex, as well geographical locations and levels of deprivation. Despite these strengths, there remain several limitations. Firstly, although this survey achieved a relatively high response rate, there is still the possibility that the sample who did participate are not precisely representative of those who did not, and of the wider population. Secondly, the standardised measures used, although well tested, were all based on parent-report or self-report, rather than clinician administered interviews, which may affect estimation of prevalence rates. Relatedly, the t-scores for the RACDS were based on data from the US, and diagnostic criteria are derived from the DSM-IV and not the DSM-5. Thirdly, although the survey was designed to collect data that would enable as comprehensive an exploration of the mental health of children and young people as possible, inevitably, not all issues could be included and even the relatively high number that were included could not be explored in substantial depth. This reflects some of the more practical and ethical considerations of the survey design, including what is a reasonable length of interview, especially for children. Fourthly, the present analysis does not assess the rates of other mental health problems that may be present in the youth population of NI or the extent to which they interact with common mental health disorders and other variables of interest. Finally, this study was cross-sectional in nature, and hence, no causal inferences can be made about the associations between the risk factors and mental health outcomes. The NIYWS provides a rich and complex dataset and it is a long-term goal of the research team to provide these estimates, and expand on the understanding of mental health in the young people of NI.

This study provides the first report of the current prevalence estimates and associated correlates of common mood and anxiety disorder in the NI youth population, enabling broad comparisons with prevalence findings from other countries. Given NI’s history of armed conflict and high levels of deprivation and economic adversity, it would seem to have all the ingredients to produce high levels of mood and anxiety disorders in youth. The NIYWS produced an overall prevalence rate of 11.5%, a somewhat higher figure than reported internationally or in England (Polanczyk et al., 2015; Vizard et al., 2018), but reflective of the findings from research with adults which has shown Northern Ireland to have 25% higher rates of common mental health disorders than other parts of the UK. England (Bunting, et al., 2012. McManus et al., 2016). Despite the somewhat elevated prevalence rates, the data from this study suggests that there is a detectible resilience in the youth of NI, with the vast majority not meeting the criteria for a common mental health disorder. Nevertheless, there is no room for complacency and the findings point to the need to develop policy and service provision in a number of areas.

The strong association between poor child health, special educational need status and common mood and anxiety disorders highlights the importance of an inclusive and integrated approach to mental health policy and provision that involves not just the healthcare sector, but education and disability sectors also. The processes already in place for assessing children with special educational needs should reflect the increased risk of mental health problems evident amongst this group and incorporate mechanisms for on-going monitoring and support. Although area-level deprivation was not significantly associated with common mood and anxiety disorders when controlling for other factors, the fact that living in a household in receipt of benefits remained significant also has potential implications for anti-poverty policy. Again, the education sector has an important role to play and the results suggest that both children in receipt of free schools meals, and those with special educational needs, are key target groups for future service development.

Likewise, the association between parental mental health and child mental health points to the need for family friendly mental health services, ensuring that primary care mental health practitioners take into account, and provide support for, the children of mental health care recipients. The pandemic has further compounded young people’s difficulties in maintaining good mental health with an increase in children reporting anxiety and depression attributed to social isolation from their peers and wider family members, as well as lack of respite from the stresses of normal family life (Prime et al., 2020). Importantly, children of parents with a mental health problem parent may have been more exposed to this and may also have had to take on more responsibility for caring for their parent in the absence of face to face mental health service provision. In meeting these increased needs, there needs to be greater recognition that mental health difficulties affect the whole family system and an associated shift from an individual level response to a family level response.
